# Different types of cluster membership in parallel-group cluster-randomised trials, where the clusters are institutions: a classification system to aid identification, with six proposed designs

**DOI:** 10.1186/s13063-025-09066-4

**Published:** 2025-09-29

**Authors:** L. E. Marsden, C. A. Surr, A. W. Griffiths, A. J. Farrin, A. J. Copas, R. E. A. Walwyn

**Affiliations:** 1 Leeds Institute of Clinical Trials Research, University of Leeds, Worsley Building, Clarendon Way, Leeds, LS2 9JT UK; 2 Centre for Dementia Research, Leeds Beckett University, Leeds, UK; 3 Institute of Population Health, University of Liverpool, Liverpool, UK; 4 MRC Clinical Trials Unit, University College London, London, UK

**Keywords:** Randomised controlled trial, Parallel group design, Cluster randomisation, Closed-cohort, Cross-sectional, Open-cohort, Continuous recruitment, Variable exposure duration

## Abstract

**Background:**

Four main types of cluster-randomised trial (CRT) are well known: parallel-group (PG), factorial, stepped-wedge and crossover designs. This established typology relates to how clusters are exposed to intervention(s) or control(s) during the trial. Published guidance is lacking on how to link design features to how individuals within clusters may be exposed and measured. Thus, the aim of this paper was to develop a classification system for different types of cluster membership in CRTs, focussing on PG designs and building on our experiences of delivering a care home trial.

**Methods:**

The classification system was developed in seven stages: (i) a scoping review was conducted to explore the use of open-cohort PG-CRTs in a range of settings; (ii) a version of the classification system was developed, using the stepped-wedge CRT typology; (iii) this was tested using a sample of published trials from the scoping review; (iv) a second version was developed, reviewed and further amendments made to aid clarity; (v) 15 trialists with experience of CRTs in a range of settings provided feedback in a 1-day, face-to-face user engagement workshop; (vi) a wider group of 39 trialists completed an online survey, providing examples and additional feedback; and (vii) all authors reviewed and approved the final version.

**Results:**

Six types of cluster membership in PG-CRTs are proposed: the closed-cohort and cross-sectional designs already established, a new-admission-continuous-recruitment, open-cohort with discrete-recruitment, open-cohort with continuous-recruitment, and a non-standard closed-cohort design. The final classification system is made up of six core design features and five additional design considerations. Diagrams of each type of cluster membership are introduced and used to illustrate examples.

**Conclusions:**

Implications of distinctions between the six types of cluster membership for the statistical analysis require further research. CONSORT guidance needs updating to include specific guidance on reporting the type of cluster membership alongside the description of how design features apply to clusters. Further methodological research is required into both the statistical and the practical implications of adopting previously unlabelled but frequently used types of cluster membership.

**Supplementary Information:**

The online version contains supplementary material available at 10.1186/s13063-025-09066-4.

## Background

In randomised trials, groups of individuals such as patients in hospitals, children in schools, residents in care homes or whole communities are often referred to as ‘clusters’. In cluster-randomised trials (CRTs), the clusters, rather than the individuals, are randomised, so clusters of individuals are the experimental units rather than the individuals [1]. There are four main types of CRT design (see [2] for an illustration): these are parallel-group (PG), factorial, stepped-wedge and crossover designs. In PG-CRTs, clusters are randomised to an intervention or a control. In factorial CRTs, clusters are randomised to a combination of the levels of two or more treatment factors. In stepped-wedge CRTs, clusters are randomised to the timing of when they switch from control to intervention. In crossover CRTs, clusters are randomised to whether they receive an intervention prior to, or following, a control. These distinctions relate to how clusters are exposed to an intervention or control over time but are not sufficient to fully describe the design of a CRT.

Within each CRT design, there exists a second typology for how and when individuals within clusters are exposed to intervention or control, and for when data on individuals is collected. We refer to this second typology as the ‘types of cluster membership’. Specifying the types of cluster membership enables the relevance of the CRT design to the research question (and specific estimands of interest) to be more fully considered, the CRT to be more easily replicated, and its primary analysis to follow more completely from its design. It also facilitates easier identification of a CRT design for reviewing purposes and highlights areas requiring further methodological development. This second typology is currently most clearly developed for stepped-wedge CRTs [3]. Copas et al. [3] identified three main designs: closed cohort, open cohort and continuous recruitment short exposure. They also identified key features that vary across these designs, enabling stepped-wedge CRTs to be classified: (i) timing of the start of exposure to the intervention, (ii) duration of exposure and (iii) measurement of outcomes. Examples of each stepped-wedge CRT design are found in the literature [4–6]. Having a classification system is important to support detailed description of trial methods in publications. It could equally serve as a checklist when considering components of the trial design at the planning stage.

A comprehensive typology for cluster membership, and a system to support classification, does not currently exist for PG-CRTs. A consequence is that many PG-CRTs fail to clearly report this aspect of design [7]. This may follow from the CONSORT extension to CRTs [8] only advising trialists to include a ‘description of how design features apply to clusters’, with no reference to cluster membership. Two types of cluster membership are well-established [9–11]: closed cohort, where individuals in clusters are recruited prior to cluster randomisation and then followed up at all measurement points, and cross-sectional, where potentially non-overlapping individuals are sampled at each measurement point. Other types exist, however, and some are commonly used but remain largely unlabelled and under-researched [7]. One is the open cohort design, where individuals in clusters are recruited prior to cluster randomisation, but also following randomisation, and are followed up (potentially repeatedly) at subsequent measurement points (see [12]). The aim of this paper was therefore to develop a classification system for PG-CRTs to aid identification of the type of cluster membership.

## Motivating example

The DCM-EPIC CRT [13, 14] highlighted the need for greater recognition that there are more than two types of cluster membership possible for PG-CRTs (see Table 1). If this had been appreciated at the design stage, it would have avoided the need to change the trial design mid-trial.

**Table 1:** Motivating example: DCM-EPIC

DCM-EPIC compared the clinical and cost-effectiveness of an intervention to improve the uptake of person-centred care (Dementia Care Mapping, DCM) plus usual care to usual care alone for people living with dementia in UK care homes. The intervention was targeted at the care home as a whole, but the primary outcome (agitation) was assessed in residents with dementia, 16 months following care home randomisation. Initially, a closed cohort PG-CRT design was adopted, with residents with dementia registered prior to cluster randomisation (baseline) and followed up at 6 and 16 months following cluster randomisation. However, unavoidably high loss to follow-up due to resident death or movement out of the care home, led to a design change to additionally include those residents with dementia at 16 months following randomisation not included at baseline. This allowed us to consider the open population of people living with dementia in UK care homes from baseline to final follow-up, who were exposed to the care home level intervention. There was substantial overlap in the residents included at baseline and final follow-up, distinguishing this open cohort PG-CRT design from a repeated cross-sectional PG-CRT design. Although linkage of some data over time within residents would have been possible, this was not done in the primary analysis	

The challenges raised by this example are common to all PG-CRTs, but if clusters are communities or geographical areas, further challenges may be faced around sampling of individuals in clusters, not as frequently faced in other settings (see Kasza et al. [12] for more details). As a result, the focus of this paper is on PG-CRTs where the clusters are institutions (e.g. care homes, schools, hospitals, prisons).

## Methods

The following six stages were used to develop the classification system. First, author LEM conducted a scoping review [7] to explore the use of open cohort PG-CRTs in a range of settings. Second, authors LEM and REAW drafted an initial version of the classification system, using the stepped-wedge CRT system [3] and our experience with the DCM-EPIC trial [13, 14] as a starting point. Third, author LEM tested the initial draft of the classification system using a sample of published trials from the scoping review [7], iteratively making amendments to widen its applicability. Fourth, authors REAW and AJF reviewed the second draft of the classification system and made further amendments to aid clarity. Fifth, in a 1-day, face-to-face, user engagement workshop (see [15] for more details), which took place in October 2019, authors LEM, REAW, CAS, AWG and AJF asked 15 trialists with experience of CRTs in a range of settings for feedback on the second draft of the classification system. After an initial presentation, attendees were asked to use the classification system, diagrams of the proposed types of cluster membership and a blank template to classify their own examples. Following this, a whole-group discussion facilitated by author AJF was audio recorded and transcribed. Based on this discussion and the completed templates, author LEM made further amendments. Finally, authors LEM, RW, CAS, AWG and AJF developed an online user engagement survey to collate examples of open cohort PG-CRTs and test the third draft of the classification system with a wider group of 39 trialists. The survey was circulated to workshop attendees, clinical trial unit networks, statistical mailing lists, members of UK medical funding panels, chief investigators of current and recently published relevant CRTs and advertised at conferences and via social media throughout 2020. All participants had to have been involved in a PG-CRT where an intervention was targeted at a cluster level. Responses to each survey item were considered by authors LEM and REAW, with final amendments made by author LEM when the survey responses indicated further points or clarification was needed. All authors reviewed and approved the final version.

## Results

### A typology of six types of cluster membership

**Fig. 1:**
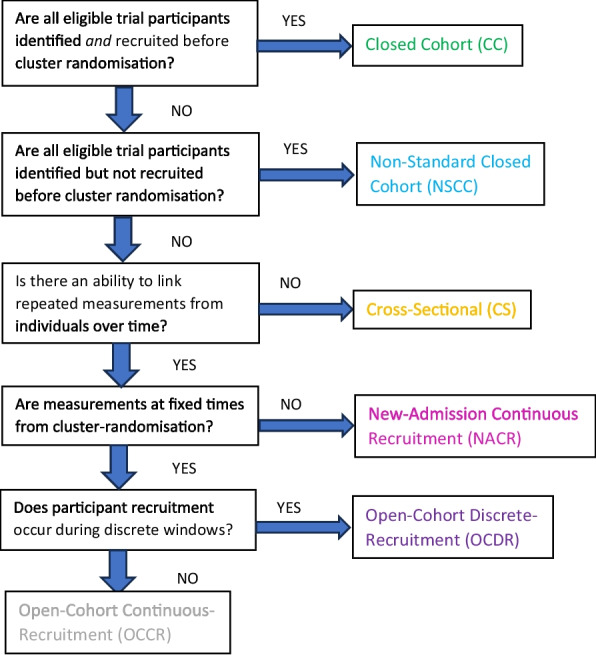
Flowchart identifying PG-CRT designs

Six sub-designs are proposed in the context of institutional PG-CRTs: the closed cohort (CC) and (repeated) cross-sectional (CS) designs already established [9–11], and a new-admission continuous-recruitment (NACR) design, an open-cohort discrete-recruitment (OCDR) design, an open-cohort continuous-recruitment (OCCR) design and a non-standard closed cohort (NSCC) design (see Fig. 1).

In the new-admission continuous-recruitment (NACR) design, individuals in clusters are continuously recruited (passively or actively) over time following cluster randomisation as they become eligible (e.g. as individuals are newly admitted to the cluster), and measurements are taken at fixed time points relative to each individual’s date of recruitment or another time point specific to that individual. In both open-cohort designs, individuals can become eligible before and after cluster randomisation. In the open-cohort discrete-recruitment (OCDR) design, eligibility occurs at fixed times between baseline and final follow-up. In the open-cohort continuous-recruitment (OCCR) design, eligibility occurs at baseline and then continuously following cluster randomisation. In the open cohort and cross-sectional (CS) designs, measurements are taken at fixed times from cluster randomisation; so, unlike the new-admission continuous-recruitment (NACR) design, individuals will have variable periods of exposure to the trial interventions at each measurement point. In both open-cohort designs, unlike the CS design, there is the ability to link repeated measurements on individuals. In the standard closed-cohort (CC) design, all individuals are recruited prior to cluster randomisation, while in the non-standard closed cohort (NSCC) design, individuals are identified prior to cluster randomisation but their recruitment occurs after cluster randomisation. Figure 1 is intended as an aid to identifying PG-CRT designs using a minimum number of questions. The following section provides elaboration on each question, useful for complex cases.

### The classification system

**Fig. 2:**
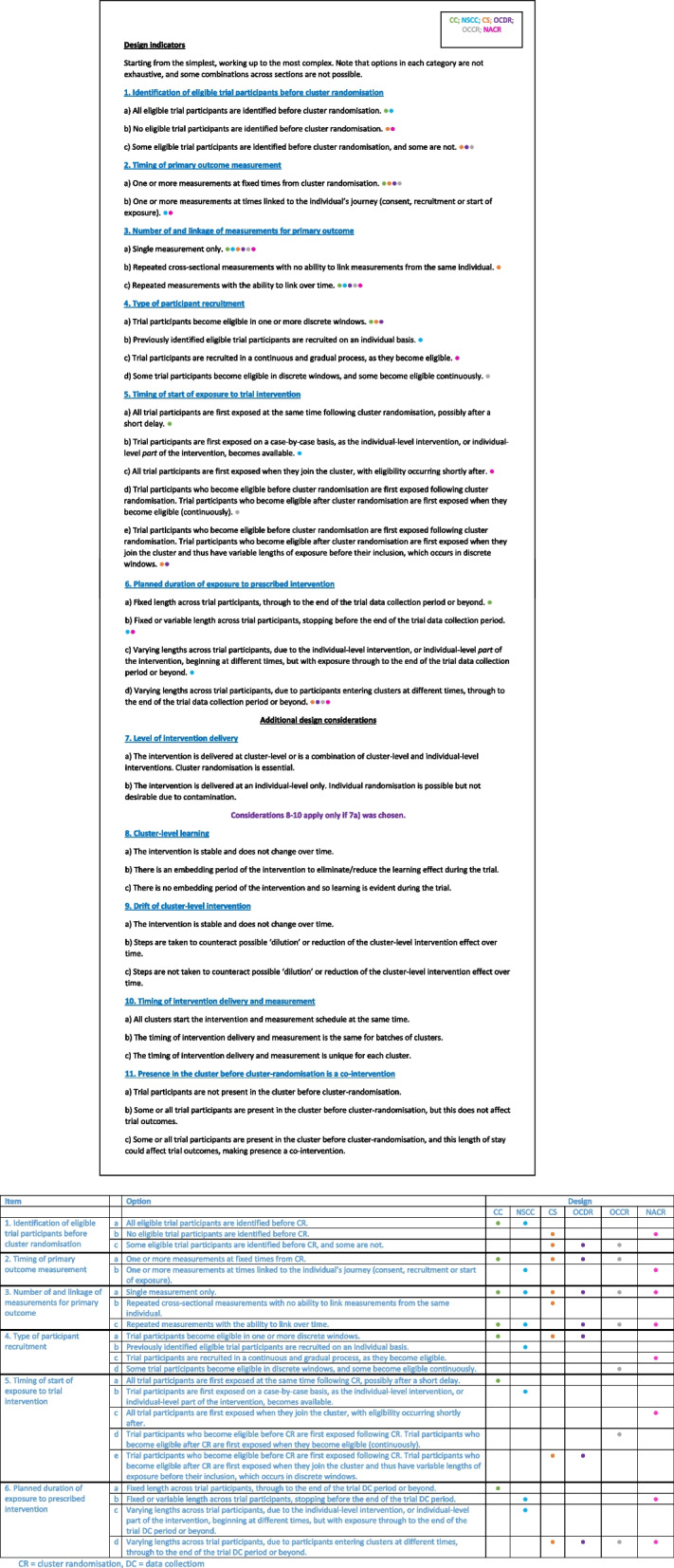
Classification of trial designs

The final classification system is made up of six core design features and five additional design considerations, making a total of eleven items (see Fig. 2).

The options for each item generally start simple and become progressively more complex. Some combinations of items are not possible. While the classification system was designed to be as general as possible, the options are not necessarily exhaustive. Note that individuals in clusters are referred to here as participants, and we focus on those measured for trial outcomes (e.g. residents) acknowledging there may be other trial participants such as health/social care staff. Note also that we make a distinction between interventions with cluster-level components (i.e. those directed at staff or services such as training) and individual-level components (i.e. direct treatment of patients). It is common that interventions have components at multiple levels (e.g. cluster and individual).

#### Core design features for cluster membership

##### Item 1: identification of eligible participants before cluster randomisation

As can be seen in Fig. 1, this item distinguishes closed-cohort (CC) designs from non-standard closed-cohort (NSCC) designs. It is also a partial indicator of whether the PG-CRT is susceptible to identification and/or recruitment bias [16–19], both of which are forms of selection bias. The timing of recruitment would also need to be known. If participants are recruited before cluster randomisation (option (a)), there is no risk of identification or recruitment bias. Option (a) paired with recruitment following cluster randomisation eliminates risk of identification bias but not risk of recruitment bias [16]. If no participants are identified before cluster randomisation (option (b)), or some are and some are not (option (c)), there is also a risk of recruitment bias. Risk of recruitment bias can be reduced or even eliminated using additional design features [16–19].

In trials where participants are not consented, identification bias can still occur if participants are identified after cluster randomisation [20]. Reference is therefore deliberately not made to consent processes in this item. For example, in a trial where participants are not aware they are part of a trial and are not contacted, identification bias can occur if the recruiter is not blinded to allocation [16]. If blinding of the identifier is not possible, identification bias could occur if the intervention improves identification skills following cluster randomisation or participants are attracted to an intervention cluster because they are seeking treatment and know it is being offered at a particular cluster [20].

##### Item 2: timing of primary outcome measurement

The timing of outcome measurement relates to the timescales that are important. If measurement timings are anchored to cluster randomisation, the cluster timescale is likely to be of interest. If they are specific to a participant, the focus shifts to the individual timescale. Use of a cluster timescale distinguishes closed-cohort (CC), open-cohort (OCDR and OCCR) and cross-sectional (CS) designs from new-admission continuous-recruitment (NACR) and non-standard closed cohort (NSCC) designs. In instances where outcomes are events that may occur at any time, and observation is passive and continuous throughout the trial, we view this as reflecting the cluster timescale. There may be cases where the timescale varies across outcomes within a trial; here we suggest focussing on the primary outcome(s). If there are co-primary outcomes on the same timescale, identification of the design will be more straightforward; if co-primary outcomes span cluster and individual timescales, other items will need to be consulted to classify the design.

##### Item 3: number of and linkage of measurements for primary outcome

The ability to link repeated measurements from participants over time distinguishes the cross-sectional (CS) design from the others. In a cross-sectional (CS) design, health or social care systems data may be used, or the participants’ identity may not be recorded or known. The phrasing of 3b and 3c with ‘ability to link over time’ is intended to make it clear that linkage at the analysis stage will not be possible when adopting a cross-sectional (CS) design. Even if linkage is possible, however, it may not be done. An open-cohort (OCDR or OCCR) design might have a cross-sectional analysis if linkage data are available but not used.

##### Item 4: type of participant recruitment

By participant recruitment, what is often implied is consent for data collection rather than consent for randomisation or intervention exposure, as the latter often occurs at a cluster level [21]. For our purposes, what informs whether the recruitment process is discrete or continuous is the timing by which participants become eligible rather than the window in which consent is undertaken. For example, participants may be recruited over a fixed period but become eligible as they join a cluster. If the participants become eligible in continuous time (that is, on a daily basis), we would regard recruitment to be continuous not discrete. The type of participant recruitment distinguishes the new-admission continuous-recruitment (NACR) design from other design sub-types, but also the open-cohort discrete-recruitment (OCDR) design from the open-cohort continuous-recruitment (OCCR) design. If participants are included in a trial analysis from anonymised health or social care systems data, but are not actively recruited or consented, it is still possible that they became eligible on a discrete or continuous basis. This information would be used to inform the classification of a trial.

##### Item 5: timing of start of exposure to trial intervention

When trial participants first experience the trial intervention also distinguishes the designs. It contributes to the length of exposure to the trial intervention received, which can vary across participants. If the trial intervention is directed at an individual level only, then the accumulating duration or ‘dose’ received will most likely be anchored to the individual timescale. With a cluster-level intervention, a discrepancy may exist between the start of a participant’s exposure and their consent to data collection or baseline. This could be problematic if the dose received is anchored to the cluster timescale in the analysis. In a closed-cohort (CC) design, where all participants consent before cluster randomisation, it is not an issue. For a non-standard closed-cohort (NSCC) design, if a cluster-level intervention is rolled out once an individual-level intervention is available, following participant consent, then again it is not an issue. With continuous recruitment (new-admission continuous-recruitment (NACR) and open-cohort continuous-recruitment (OCCR)), the gap is likely to be negligible. However, in an open-cohort discrete-recruitment (OCDR) or cross-sectional (CS) design, participants could be exposed to a cluster-level intervention for a long period of time before being consented and before data collection. When this is an issue, increasing the number of recruitment points could be helpful.

##### Item 6: planned duration of exposure to trial intervention

Whether the overall planned duration of a participant’s exposure to the trial intervention is fixed or variable is the final item contributing to the classification of the designs. In a closed-cohort (CC) design, it is anticipated that participants have a fixed duration of exposure to the trial intervention from cluster randomisation to final follow-up. It is possible that the planned exposure duration is fixed in a non-standard closed-cohort (NSCC) or new-admission continuous-recruitment (NACR) design, but here participant’s exposure could end before data collection is completed. In all other cases, exposure is anticipated to continue through to the end of the trial data collection period or beyond. Variable lengths of planned (not unintentional) exposure to the trial intervention is a feature of all designs except for the closed-cohort (CC) design. A distinction is made, however, between whether the reason for variable lengths of planned exposure is that the start of an individual-level component of the intervention is staggered (the non-standard closed-cohort (NSCC) design), or that participants enter clusters at different times (cross-sectional (CS), open-cohort discrete-recruitment (OCDR), open-cohort continuous-recruitment (OCCR), new-admission continuous-recruitment (NACR) designs).

### Additional design considerations

#### Item 7: level of intervention delivery

If an intervention is delivered at an individual level, randomisation of participants to interventions may be possible but considered undesirable due to the risk of contamination within the cluster. Here, the intervention may be viewed as independent of the cluster, as presence in the cluster does not necessarily lead to direct exposure to the intervention (it may still lead to indirect exposure). If part of the intervention is delivered at a cluster level, cluster-randomisation is essential. If the intervention is delivered at an individual level, the individual timescale becomes of interest. If the intervention is delivered at a cluster level, the cluster timescale becomes of interest. There are grey areas where interventions have multiple components operating at multiple levels and a PG-CRT design is adopted. Here, multiple timescales will be of interest. This item is an additional design consideration rather than a feature of the design, because new-admission continuous-recruitment (NACR) designs do not always involve an individual-level intervention only, nor do closed-cohort (CC) designs always have to include cluster-level interventions, for example.

#### Item 8: cluster-level learning

A cluster-level intervention may be stable or learning may occur, for example, via staff increasing their expertise as they practice their skills. The trial design may allow an ‘embedding period’ of the intervention before participant exposure to allow the intervention effect to stabilise (or the intervention to become established) before outcomes are measured. Without such an embedding period, learning is possible during the trial. This has implications for the dose of the trial intervention received, through timing of participants starting to be exposed to the trial intervention.

#### Item 9: drift of cluster-level intervention

Drift may also occur if trained staff leave an institution and new staff replace them, or if staff trained at the start of an intervention period are trained once or infrequently, and elements of their training are forgotten, become less effective or adapt to their individual practice. Lack of supervision of staff and monitoring of their effectiveness can also exacerbate drift. This can lead to dilution of the intervention effect. Subsequent training for new staff or refresher training for existing staff may be introduced to mitigate drift.

#### Item 10: timing of intervention delivery and measurement

A cluster-level intervention may be delivered to all clusters at the same point in calendar time; it may be staggered and delivered to batches of clusters at each point in discrete calendar time, or the timing of intervention delivery may be unique for each cluster in continuous calendar time. This may become important if a change in policy is introduced during the trial or seasonal effects are expected, for example, and may be a reason to block cluster randomisation by calendar time. There may be similar considerations if the timing of data collection across clusters differs in calendar time.

#### Item 11: presence in the cluster before cluster-randomisation is a co-intervention

Where participants can be present in a cluster before randomisation, time-in-cluster before cluster randomisation could be seen as an intervention in its own right or a co-intervention. This might occur if presence in the cluster, irrespective of a trial intervention, can impact participant outcomes. Resident’s level of agitation may be affected by the length of time in a care home, for example. In this case, participant length of stay in the cluster might be important to consider in the analysis.

### Schematics of types of cluster membership

**Fig. 3:**
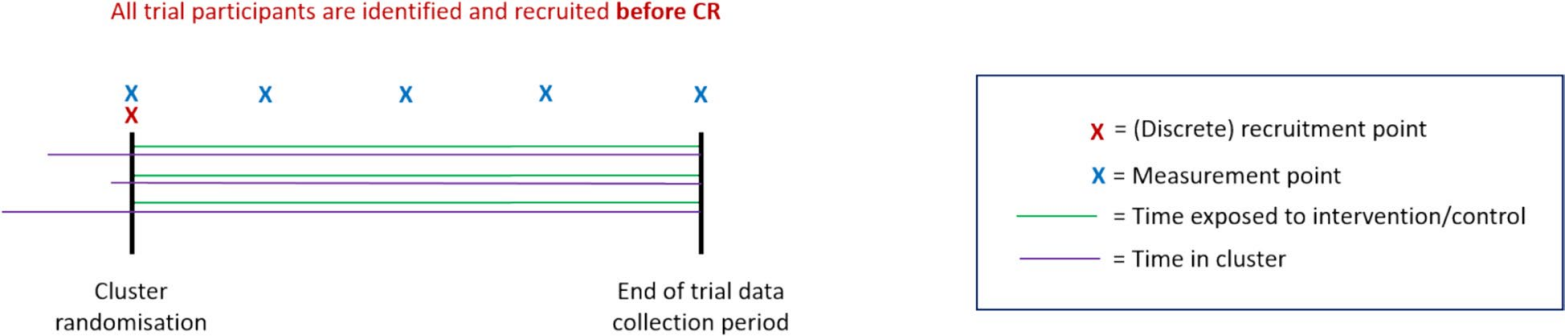
Diagram of the closed cohort (CC) design

A schematic of the closed-cohort (CC) design is given in Fig. 3. This is based on the FinCH CRT (Table 2).

**Table 2:** Falls in Care Homes (FinCH)

FinCH was a PG-CRT in care homes assessing effectiveness of a fall prevention programme [22]. All eligible participants were identified and recruited before cluster-randomisation (1a, 4a). Participants were assessed for the primary outcome 3 to 6 months following cluster-randomisation (2a, 3c). The intervention was at a cluster-level (7a) and included training of care home staff, provision of manuals and a poster displayed in care homes. All participants were exposed to the intervention at the same time following cluster-randomisation (5a) through to the end of the trial data collection period (6a). Although provision of manuals and posters were stable interventions (8a, 9a), staff training was not. Staff training had an embedding period of 3 months before outcomes were assessed (8b); refresher training was provided to counteract drift (9b). No information on the timing of intervention delivery and measurement across clusters was provided. All participants were present in the cluster before cluster-randomisation (11b/c)	

**Fig. 4:**
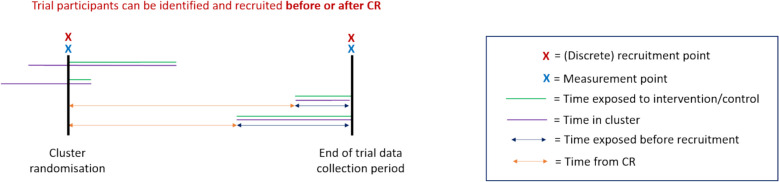
Diagram of the cross-sectional (CS) design with no overlaps

A schematic of the cross-sectional (CS) design is given in Fig. 4. This is based on the AFFINITIE CRT (see Table 3).

**Table 3:** Audit and Feedback INterventions to Increase evidence-based Transfusion practIcE (AFFINITIE)

AFFINITIE was a programme of factorial CRTs in secondary care assessing the effectiveness of two feedback interventions (audit report and support) in a national comparative audit [23]. In both trials, clusters were the transfusion practitioners in NHS trusts. Due to the audit topic, which was elective surgery, participants at baseline and follow-up were unlikely to overlap, so some eligible trial participants were identified before cluster randomisation and some not (1c). The primary outcome was assessed at 12 months following cluster-randomisation (2a). As audit data was anonymised, it was not possible to link any repeated measurements from a participant (3b). Interventions targeted staff at a cluster-level (7a). Participants became eligible in discrete windows at baseline and 12 months following cluster-randomisation (4a). Trial participants at baseline were first exposed following cluster randomisation, while those at 12 months were exposed when they are admitted to hospital (5e). As participants were exposed during their hospital stay, exposure duration across participants was variable (6b). No embedding period was implemented so cluster-level learning might have occurred (8c). There was only one round of audit and feedback so drift might have occurred (9c). Audit reports were delivered at the same calendar time for all clusters, with support available from randomisation (10a). At 12 months trial participants were unlikely to have been present in the hospital before cluster randomisation (11a)	

**Fig. 5:**
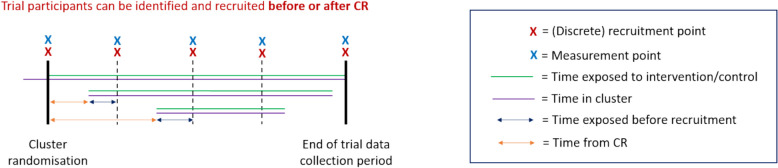
Diagram of the open-cohort discrete-recruitment (OCDR) design

A schematic of the open-cohort discrete-recruitment (OCDR) design is given in Fig. 5. This is based on the SEHER CRT (see Table 4). Note that extra measurement and recruitment points have been included in Fig. 5 to show the possibilities for this design. With only 2 recruitment points, the time exposed before recruitment represented by blue arrows would be considerably larger.

**Table 4:** Strengthening Evidence base on scHool-based intErventions for pRomoting adolescent health (SEHER)

SEHER was a PG-CRT in secondary schools assessing the effectiveness of an intervention to improve school climate and health outcomes [24]. Students in school at baseline and follow-up (or both) were eligible, so some trial participants were identified before cluster randomisation and some not (1c). The primary outcome was assessed on students 8 months after cluster-randomisation (2a). Some students were in the school at both baseline and follow-up, so it was possible to link repeated measurements (3c). The intervention was multi-component, consisting of cluster and individual-level components (7a). Students were consented at baseline and follow-up in two discrete windows (4a). Students in school at baseline were exposed to the intervention following cluster-randomisation; students enrolling later were exposed when they joined the school but were not recruited until 8-month follow-up (5e). As such, there were variable exposure lengths across students (6d). Due to a pilot before the main trial, the intervention was already embedded to some degree (8b). Training and supervision of counsellors and teachers was provided throughout to counteract possible drift (9b). Information on the timing of intervention delivery and measurement across clusters was not reported. Some students were in school before cluster-randomisation, so their time-in-school before cluster-randomisation could affect trial outcomes (11b/c)	

**Fig. 6:**
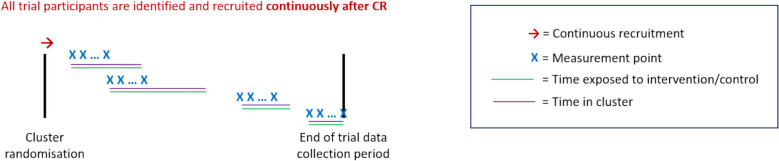
Diagram of one example of the new-admission continuous-recruitment (NACR) design

A schematic of the new-admission continuous-recruitment (NACR) design is given in Fig. 6. This is based on the POD CRT (see Table 5). Note that other variants of a new-admission continuous-recruitment (NACR) design are possible which allow a participant’s exposure period to be fixed rather than variable (6b) and continue to the end of the trial data collection period (6d) (see Appendix for examples).

**Table 5:** Prevention Of Delirium (POD)

POD was a feasibility PG-CRT in orthopaedic trauma wards of an intervention to prevent delirium in elderly care [25]. Participants became eligible when admitted to a ward, so no eligible trial participants were identified before cluster randomisation (1b). Data collection was post-admission and was therefore unique to each participant (2b). The primary outcome was assessed over 10 days of hospital admission; repeated measurements were obtained with the ability to link outcomes over time (3c). POD was a ward-based intervention involving staff and volunteers to change the environment experienced by participants (7a). Trial participants were recruited continuously, as they became eligible (4c). As a result of the intervention level, participants were first exposed when they joined the ward, and if they met eligibility criteria were asked to consent within 48 h of admission (5c). Participants were then exposed until they were discharged (6b). Following cluster randomisation, intervention wards underwent a 6-month embedding period before participants were recruited (8b). No information could be found about refresher training for staff in the ward or training for new staff (9a/b/c). All wards began participant recruitment at the same time (10a). Participants were not present in the ward before cluster-randomisation, so time in the ward at baseline was not an issue (11a)	

**Fig. 7:**
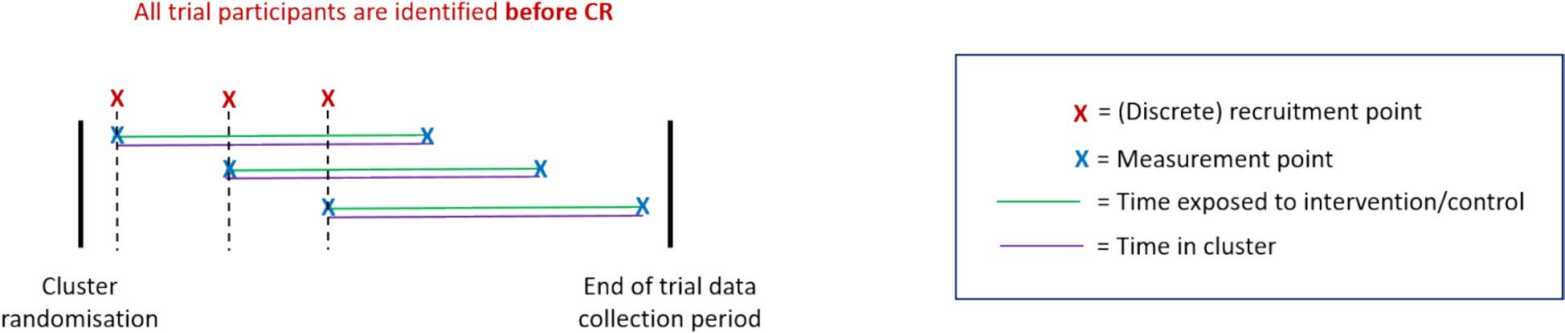
Diagram of the non-standard closed cohort (NSCC) design

A schematic of the non-standard closed-cohort (NSCC) design is given in Fig. 7. This is based on the PROSPER CRT (see Table 6). Note the similarities between a non-standard closed-cohort (NSCC) design and a new-admission continuous-recruitment (NACR) design. The main difference is in the type of participant recruitment (4b versus 4c).

**Table 6:** PeRsOnaliSed Care Planning for OldER People (PROSPER)

PROSPER was a feasibility PG-CRT in general practices, assessing whether a care planning intervention could improve frailty in older people [26]. All eligible trial participants were identified before cluster randomisation (1a). Participants were assessed for the primary outcome at 12 months following consent (2b, 3c). A team-based intervention was delivered to general practices (7a). Participants who had previously been identified before cluster randomisation were consented after cluster randomisation on an individual basis for logistical reasons (4b). Participants were exposed on an individual basis following consent (5b). The intervention was a fixed duration of 12 weeks (6b)	

**Fig. 8:**
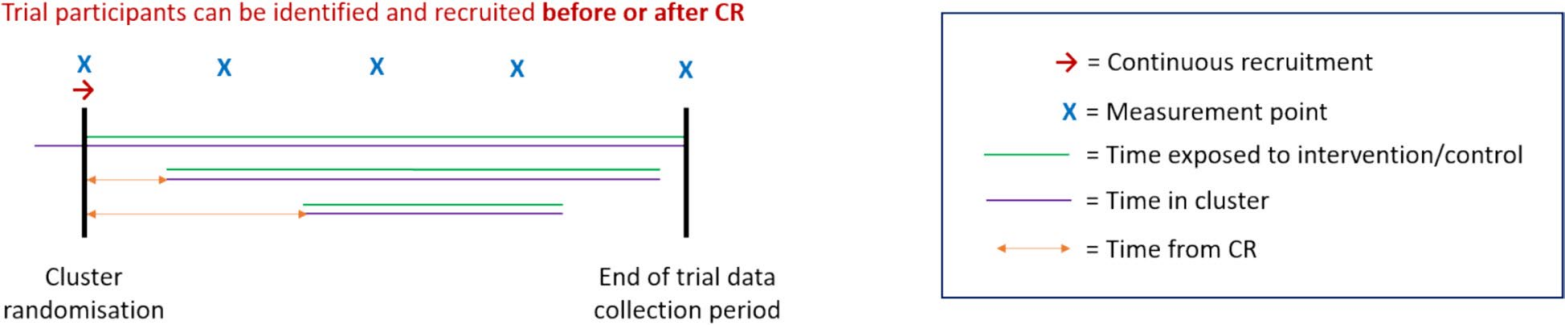
Diagram of the open-cohort continuous-recruitment (OCCR) design

A schematic of the open-cohort continuous-recruitment (OCCR) design is given in Fig. 8. This is based on the VIVALDI-CT CRT (see Table 7).

**Table 7:** Shaping care home COVID-19 testing policy (VIVALDI-CT)

VIVALDI-CT is a PG-CRT in care homes, assessing the impact of regular asymptomatic testing of care home staff for COVID-19, compared to national testing guidance, on incidence of COVID-19-related hospital admissions in residents [27]. Some residents are identified before cluster randomisation, while some are not (1c). The primary outcome is anchored to the randomisation of care homes (2a) with constant surveillance for hospital admission events in residents thereafter (3c). Although the intervention is delivered to care home staff, to achieve its aims it is delivered to all staff making cluster randomisation essential (7a). Some residents become eligible at baseline, while others become eligible as they enter the care home thereafter (4d). Residents who enter the care home prior to cluster randomisation are first exposed to the intervention following cluster randomisation, while those who enter the care home after cluster randomisation are first exposed when they enter the home (5d). As such, there are variable exposure lengths across residents (6d). While there is no embedding period for the intervention, it is not clear whether learning effects are likely (8a/c). Similarly, it is not clear what strategies were considered to counteract possible drift (9a/c). All care homes will be randomised at the same time, or in a phased approach by different providers as they become ready (10a/b). Some residents were in the care home before cluster randomisation, so their length of stay could affect trial outcomes (11b/c)	

## Discussion

In our proposed classification system, we have highlighted the key features of six proposed types of cluster membership and clearly described what differentiates them. While closed-cohort (CC) and cross-sectional (CS) designs have been defined previously [9–11], the open-cohort discrete-recruitment (OCDR), open-cohort continuous-recruitment (OCCR), new-admission continuous-recruitment (NACR) and non-standard closed cohort (NSCC) designs are defined here for the first time. The distinction of whether participants are recruited in discrete time intervals (CC, CS, OCDR) or continuously (OCCR, NACR, NSCC) is also new. While Copas et al. [3] link the length of the exposure period to continuous recruitment for stepped wedge CRTs, we define three distinct designs that involve continuous recruitment, including broad NACR and NSCC designs with different types of exposure period possible. A short exposure may mean participants are exposed to only one of two possible conditions in stepped-wedge CRTs, a consideration we believe is less important in PG-CRTs. In PG-CRTs, a more important consideration is whether the planned exposure duration (until primary outcome is ascertained) is fixed for participants (CC, NACR, NSCC) or variable across participants (CS, OCDR, OCCR, NACR, NSCC). We have argued that whether the timing of outcome measurement is linked to cluster randomisation (CC, CS, OCDR, OCCR) or an individual pathway (NACR, NSCC) links to when cluster, individual or both timescales are of interest. The implications of these distinctions for the selection of the most appropriate estimand and for the statistical analysis of PG-CRTs require further research.

It is important to acknowledge that for a particular trial context, the nature of the clusters and the intervention do partly determine the type of cluster membership, leaving trialists with only a limited choice. For example, as care homes are inherently ‘open-cohort’ clusters, with residents leaving and joining over time, the cluster membership will not typically follow a closed-cohort design for trials in care homes unless the trial time frame is short. An intervention targeted to individuals as they join a cluster would suit a new-admission continuous-recruitment design, and conversely, an intervention expected to influence all members of a cluster, for example, by changing the culture or environment, would suit an open-cohort or cross-sectional design. Methodological research is needed to provide guidance on the choice of cluster membership in the cases where trialists do have a choice. This will need to address pragmatic considerations, the ability to directly estimate the estimand of interest, and the number of clusters, participants or measurements required to do so with adequate power.

One of the strengths of the proposed classification system is its potential for improving the reporting of PG-CRT designs in future protocol and results papers. To facilitate this, we would recommend that the CONSORT statement extension for CRTs [7] is updated to include specific guidance on reporting which of the six types of cluster membership is being adopted, in addition to the six design features that feed into this, to provide further clarity. Widespread use of the diagrams introduced here in protocol papers would also aid transparency of reporting, with these diagrams providing additional detail. Finally, we recommend that the five additional design considerations are included at a minimum in protocol papers to ensure that their implications are incorporated in the planning stages. We recognise that these additional design considerations are linked to the description of the interventions and so might also come under the TIDieR checklist [28], a tool to aid reporting of complex interventions. A second strength of the proposed classification system is that it provides the framework for previously unlabelled designs frequently used in health and social care research. This aids identification of opportunities for methodological research into each type of cluster membership and its associated statistical analyses. It also facilitates discussion of some of the lesser-known biases found in each design, building on Caille et al. [17], who developed a graphical tool to improve reporting and assess the risk of different biases associated with PG-CRTs.

One limitation of the proposed classification system is its focus on PG-CRTs in institutional settings and in particular care home settings. It is possible that individuals may be cluster members in each of the six ways we identify for other designs such as stepped-wedge CRT and crossover CRT, but further work is needed to consider exactly how the design features and classification system would be defined in each case. Further variations of each cluster membership type may exist, or even new types entirely, but we believe this work provides a solid foundation on which to build. Finally, while a single NACR type is proposed, it is recognised that additional consideration is needed as to whether it could be further divided into multiple sub-types. If multiple NACR sub-types were to have differing implications for the estimand, statistical analysis or sample size calculation, then further work in this area is recommended.

## Conclusions

Our classification system provides a wide range of design options for PG-CRTs in institutional settings, such as care homes, specifying how individuals are cluster members. The selected design should be clearly reported in protocol and results papers. Further methodological research is required into both the statistical and the practical implications of adopting previously unlabelled but frequently used designs, leading to detailed guidance on when specific designs are most appropriate.

## Supplementary Information

Supplementary Material 1.

## Data Availability

The datasets used during the current study are available from the corresponding author on reasonable request.

## Abbreviations

AFFINITIE: Audit and Feedback INterventions to Increase evidence-based Transfusion practice
CC: Closed cohort
CONSORT: Consolidated Standards of Reporting Trials
COVID-19: Coronavirus disease 2019
CRT: Cluster-randomised trial
CS: Cross-sectional
DCM-EPIC: Dementia Care Mapping to Enable Person-centred care In Care homes
FinCH: Falls in Care Homes
NACR: New-admission-continuous-recruitment
NSCC: Non-standard closed-cohort
OCCR: Open-cohort with continuous-recruitment
OCDR: Open-cohort with discrete-recruitment
PG: Parallel-group
POD: Prevention Of Delirium
PROSPER: PeRsOnaliSed Care Planning for OldER People
SEHER: Strengthening Evidence base on scHool-based intErventions for pRomoting adolescent health
TIDieR: Template for Intervention Description and Replication
UK: United Kingdom
VIVALDI-CT: Shaping care home COVID-19 testing policy
