# Supplementary material for: Different types of cluster membership in parallel-group cluster-randomised trials, where the clusters are institutions: a classification system to aid identification, with six proposed designs
Source: Trials. 2025 Sep 29;26:380. doi: 10.1186/s13063-025-09066-4 (PMC12482407; doi:10.1186/s13063-025-09066-4)

**APPENDIX.**

Figure A1 - Diagram of the NACR design in the MINT trial. The grey dots represent the measurements taking place long after the exposure period.


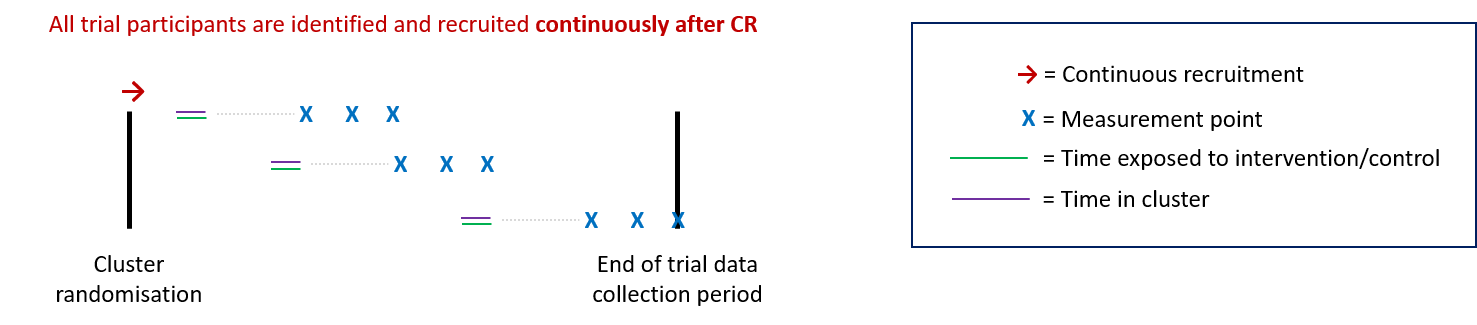


Figure A2 - Diagram of the NACR design in the PEARL trial.


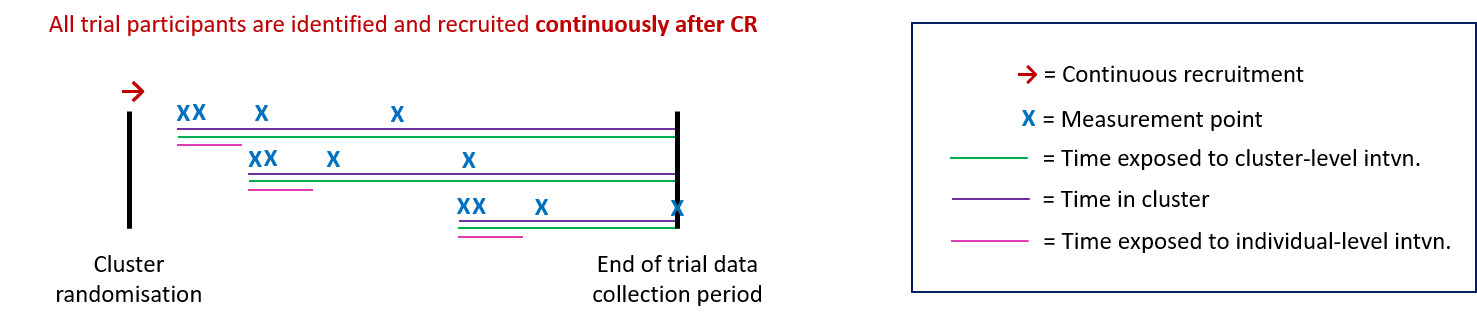

Supplement: Supplementary file 1 — Supplementary Material 1. [file 13063_2025_9066_MOESM1_ESM.docx]
